# Recent Status of Pregnancies at an Advanced Maternal Age at a Japanese Perinatal Center

**DOI:** 10.7759/cureus.35875

**Published:** 2023-03-07

**Authors:** Shunji Suzuki

**Affiliations:** 1 Obstetrics and Gynecology, Japanese Red Cross Katsushika Maternity Hospital, Tokyo, JPN

**Keywords:** assisted reproductive technology, jaan, postpartum hemorrhage, advanced maternal age, pregnancy

## Abstract

Objective

We compared the recent obstetric outcomes of women aged 40 and over as advanced maternal age (AMA) to those with AMA more than 10 years ago.

Methods

This was a retrospective study of primiparous singleton pregnancies who delivered at ≥22 weeks of gestation managed at Japanese Red Cross Katsushika Maternity Hospital between 2003-2007 and 2013-2017.

Results

The percentage of primiparous women aged with AMA who delivered at ≥22 weeks of gestation increased from 1.5 to 4.8% (p<0.01) due to the increase in the pregnancy conceived by in vitro fertilization (IVF). In the pregnancies with AMA, the percentage of cesarean delivery decreased from 51.7 to 41.0% (p=0.01), while the prevalence of postpartum hemorrhage increased from 7.5 to 14.9% (p=0.01). The latter was associated with an increased rate of in vitro fertilization (IVF) use.

Conclusions

The percentage of AMA pregnancies significantly increased with the development of assisted reproductive technology, and the prevalence of postpartum hemorrhage also increased in AMA pregnancies.

## Introduction

In the last decades, the rate of pregnancies at an advanced maternal age (AMA) has increased steadily [1-3]. This trend has seemed to be most commonly attributed to older primigravid women who delay childbearing by lifestyle choice or due to underlying subfertility [3]. In Japan, it has also been presumed to be due to the trend of late marriage accompanying women's social advancement and the development of assisted reproductive technology (ART) [4]. However, AMA, especially for ages over 40, has been reported to be an independent risk factor for adverse obstetric outcomes [1,5-8]. Most studies have agreed that AMA is related to an increase of adverse maternal and fetal outcomes such as preeclampsia, fetal growth restriction, prematurity, and cesarean delivery [1,5-8].

To date, however, it has not been sufficiently verified whether these adverse obstetric outcomes have changed. In this study, we compared the recent obstetric and perinatal outcomes of women with AMA to those more than 10 years ago.

## Materials and methods

The protocol for this study was approved by the Ethics Committee of the Japanese Red Cross Katsushika Maternity Hospital in 2021 (K2021-29).

**Figure 1:**
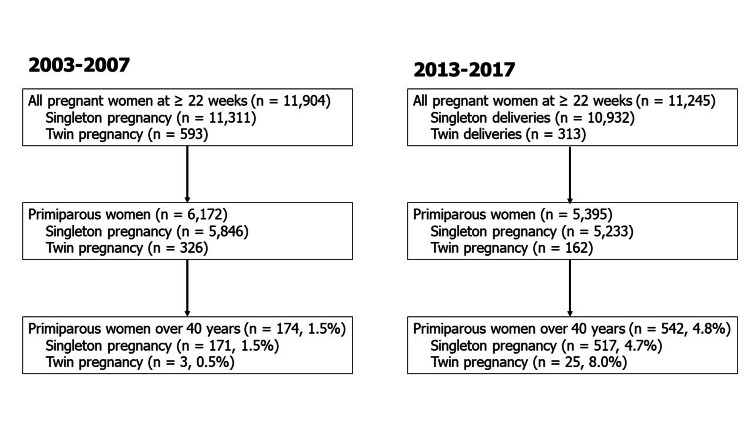
Flow diagram of study inclusion

This was a retrospective study of primiparous singleton pregnancies who delivered at ≥22 weeks of gestation managed at Japanese Red Cross Katsushika Maternity Hospital between 2003-2007 and 2013-2017. In some literature, age more than 35 years may be considered under high-risk pregnancy [2,3]; however, in this study, we defined 40 years or older as AMA because more than 30% of women undergoing fertility treatment in Japan have started the treatment after the age of 40 [9]. The periods were chosen because there were no institute relocations or renovations, and there were no restrictions on the acceptance of medical care, so it was thought that there would be no bias in data collection. A flow diagram of study inclusion is shown in Figure 1. In this study, the obstetric and perinatal outcomes in the Japanese primiparous women aged 40 years and older at delivery in 2013-2017 (n=542: recent AMA group) were compared with those in 2003-2007 (n=174: previous AMA group).

Data included antenatal data, gestational age at delivery, mode of delivery, birth weight, Apgar score at one and five minutes, neonatal intensive care unit (NICU) admission, neonatal death, and postpartum hemorrhage.

During these two periods, there were not any significant changes in the medical situation of the NICU in our institute.

Perinatal death was defined as intrauterine fetal demise at ≥22 weeks' gestation, stillbirth with the fetus weighing 500 g, and neonatal death in the first week of life. Neonatal asphyxia was defined as a neonatal Apgar score at one or five minutes of <7. Postpartum hemorrhage was defined as an estimated blood loss of ≥1,000 mL. Preterm delivery was defined as delivery at <37 weeks' gestation. In most cases, the gestational age was defined based on ultrasonography at 9-11 weeks of gestation. In cases with a delayed first visit, the gestational age was confirmed based on a neonatal neurological assessment.

Data are expressed as average and number (percentage). SPSS Statistics software version 20 (IBM Inc., Armonk, New York) was used for statistical analyses. A test of normality between the two groups was also performed. For statistical analysis, the χ2 test for categorical variables was used. Student's t-test was used for continuous variables. A p-value of <0.05 was considered significant. Odds ratios (ORs) and 95% confidence intervals (CIs) were calculated. To examine the antenatal data with obstetric and perinatal outcomes, a multivariate logistic regression analysis was conducted.

## Results

As shown in Figure 1, the percentage of primiparous women aged 40 and over who delivered at ≥ 22 weeks of gestation increased from 1.5 to 4.8% (p<0.01).

Table 1 shows the antenatal data of the clinical characteristics of the women aged 40 and over who delivered at ≥22 weeks of gestation in 2003-2007 and those in 2013-2017. The percentage of women that conceived by in vitro fertilization (IVF) increased from 20.7 to 58.1% (p<0.01). Table 2 shows the obstetric outcomes of the women aged 40 and over who delivered at ≥22 weeks of gestation in 2003-2007 and those in 2013-2017. The pregnancies with IVF use had a significantly increased risk of postpartum hemorrhage compared with those conceived spontaneously (17.1 vs. 9.3 %, crude OR 2.00, 95% CI 1.3-3.1, p<0.01). Of the 94 pregnancies with postpartum hemorrhage, 60 (63.8%) were conceived by IVF. Table 3 is an additional table showing the obstetric outcomes of these women classified into singleton and twin pregnancies. The percentage of cesarean delivery decreased from 51.7 to 41.0% (p=0.01), while the prevalence of postpartum hemorrhage increased from 7.5 to 14.9% (p=0.01). Using a multivariate analysis, the percentage of cesarean delivery decreased independently (adjusted OR 0.57, 95% CI 0.32-0.99, p=0.03), while the increased rate of postpartum hemorrhage was associated with the increased rate of IVF use.

**Table 1:** Antenatal data of the clinical characteristics of the women aged 40 and over who delivered at ≥22 weeks of gestation in 2003-2007 and those in 2013-2017 Data are expressed as average, range or number (percentage) IVF - in vitro fertilization

	2003-2007	2013-2017	p-value
Total number	174	542	
Maternal age (years)			
Average	41.1	41.4	0.08
Range	40-46	40-49	
History of IVF use	36 (20.7)	315 (58.1)	< 0.01
Twin pregnancy	3 (1.7)	21 (3.9)	0.26

**Table 2:** Obstetric outcomes of the women aged 40 and over who delivered at ≥22 weeks of gestation in 2003-2007 and those in 2013-2017 Data are expressed as number (percentage) *Women in 2013-2017 vs. those in 2003-2007 NICU - neonatal intensive care unit

	2003-2007	2013-2017	p-value*
Total number	174	542	
Preeclampsia	17 (9.8)	57 (10.5)	0.78
Preterm delivery	15 (8.6)	37 (6.8)	0.43
Cesarean delivery	90 (51.7)	222 (41.0)	0.01
Total blood loss >1,000 mL	13 (7.5)	81 (14.9)	0.01
Fetal demise	0 (0)	1 (0.2)	0.57
Live birth	177 (100)	566 (99.8)	0.57
Low birth weight infant	34 (19.5)	100 (18.5)	0.75
Light for gestational age infant	19 (10.9)	43 (7.9)	0.22
Neonatal asphyxia	4 (2.3)	6 (1.1)	0.24
Umbilical artery pH <7.0	0 (0)	0 (0)	1
NICU admission	18 (10.1)	57 (10.0)	0.97
Chromosomal abnormality	2 (1.1)	2 (0.4)	0.21
Neonatal death	0 (0)	0 (0)	1

**Table 3:** Obstetric outcomes of the women aged 40 and over who delivered at ≥22 weeks of gestation classified into singleton and twin pregnancies in 2003-2007 and those in 2013-2017 Data are expressed as average, range or number (percentage) NICU - neonatal intensive care unit

	2003-2007			2013-2017		
	Singleton pregnancy	Twin pregnancy	Total	Singleton pregnancy	Twin pregnancy	Total
Total number	171	3	174	517	25	542
Preeclampsia	17 (9.9)	0 (0)	17 (9.8)	54 (10.4)	3 (12)	57 (10.5)
Preterm delivery	14 (8.2)	1 (33)	15 (8.6)	26 (5.0)	11 (44)	37 (6.8)
Cesarean delivery	87 (60.9)	3 (100)	90 (51.7)	198 (38.3))	24 (96)	222 (41.0)
Total blood loss >1,000 mL	13 (7.6)	0 (0)	13 (7.5)	69 (13.3)	12 (48)	81 (14.9)
Fetal demise	0 (0)	0 (0)	0 (0)	1 (0.2)	0 (0)	1 (0.2)
Live birth	171 (100)	6 (100)	177 (100)	516 (99.8)	50 (100)	566 (99.8)
Low birth weight infant	30 (17.5)	4 (67)	34 (19.5)	69 (13.4)	31 (62)	100 (18.5)
Light for gestational age infant	17 (9.9)	2 (33)	19 (10.9)	32 (6.2)	11 (44)	43 (7.9)
Neonatal asphyxia	4 (2.3)	0 (0)	4 (2.3)	2 (0.4)	4 (8)	6 (1.1)
Umbilical artery pH <7.0	0 (0)	0 (0)	0 (0)	0 (0)	0 (0)	0 (0)
NICU admission	14 (8.2)	4 (67)	18 (10.1)	29 (5.6)	28 (56)	57 (10.0)
Chromosomal abnormality	2 (1.1)	0 (0)	2 (1.1)	2 (0.4)	0 (0)	2 (0.4)
Neonatal death	0 (0)	0 (0)	0 (0)	0 (0)	0 (0)	0 (0)

## Discussion

In this study, the percentage of AMA pregnancies significantly increased with the development of ART; however, the prevalence of postpartum hemorrhage in AMA pregnancies also increased under these circumstances despite the declined rate of cesarean delivery. On the other hand, the number of twin pregnancies increased; however, the perinatal outcomes did not change significantly.

Although the current study was a small one from one institute, this may be the first study that has not been examined in the past.

ART singleton pregnancies have been reported to be associated with higher risks of adverse obstetric outcomes such as postpartum hemorrhage, especially in cases with the use of frozen embryo transfer [10-13]. The increased prevalence of postpartum hemorrhage in cases of ART has been suggested to be the associations with endometriosis and hormone treatments leading to the presence of suboptimal endometrial function. AMA has been observed to serve as a surrogate factor for postpartum hemorrhage due to the associated increased risk factors and obstetric complications [14]. The current results may indicate ART as one of the risk factors for postpartum hemorrhage reported previously [14].

Once, AMA was a risk factor for cesarean delivery [15]. The tendencies do not seem to have changed even now [16,17]. However, recently the majority of older gravidae who attempt a trial of labor, even if nulliparous, have been observed to deliver vaginally without an increase in adverse neonatal outcomes [17]. In this result, we are not aware that there have been any major changes in our clinical practice in the management of AMA deliveries or the medical situation of the NICU. AMA pregnancies had once been considered 'precious pregnancies'. However, we are aware that recently the criteria for diagnosing arrest of labor and/or non-reassuring fetal status has not distinguished them from pregnancies without AMA. There may have been a change in our consciousness without a clear basis. The current results are presumed to be due to the fact that a cesarean section is no longer an easy option for AMA pregnancies. Future studies may be needed to determine whether the trends have changed other perinatal outcomes.

We understand there are some serious limitations apart from the small sample size in this study. The quality of the data may be variable. For example, there were inevitable time-related changes over the 15 years of data collection, with differences in medical staff, approach to the diagnosis, and management of obstetric complications and procedures with the evolution of ART. In addition, we have not performed infertility treatments by ourselves in our institute, so we cannot accurately estimate the bias for the current results.

At last, we understand that the most serious limitation of this study is a study of only a single institution, as mentioned above. Therefore, further examinations are needed to confirm the possibility of the same status of AMA pregnancies being seen at other hospitals.

## Conclusions

In conclusion, the percentage of AMA pregnancies significantly increased with the development of ART, and the prevalence of postpartum hemorrhage also increased in AMA pregnancies. The results are presumed to be due to the fact that a cesarean section is no longer an easy option for AMA pregnancies. They may also indicate ART as one of the risk factors for postpartum hemorrhage. On the other hand, the number of twin pregnancies increased; however, the perinatal outcomes did not change significantly.
